# Diagnostic Sensitivity of the Revised Venous System in Brain Death in Children

**DOI:** 10.3390/tomography11030030

**Published:** 2025-03-08

**Authors:** Hasibe Gökçe Çinar, Berna Ucan, Hasan Bulut, Şükriye Yılmaz, Sultan Göncü, Emrah Gün, Pınar Özbudak, Canan Üstün, Çiğdem Üner

**Affiliations:** 1 Department of Pediatric Radiology, Ankara Etlik City Hospital, Ankara 06170, Turkey; hasibegokce.cinar1@saglik.gov.tr (H.G.Ç.); hasan.bulut2@saglik.gov.tr (H.B.); sukriye.yilmaz3@saglik.gov.tr (Ş.Y.); cigdem.uner1@saglik.gov.tr (Ç.Ü.); 2 Department of Pediatric İntensive Care, Ankara Etlik City Hospital, Ankara 06170, Turkey; sultan.goncu@saglik.gov.tr (S.G.); emrah.gun1@saglik.gov.tr (E.G.); 3 Department of Pediatric Neurology, Ankara Etlik City Hospital, Ankara 06170, Turkey; pinar.ozbudak@saglik.gov.tr (P.Ö.); canan.ustun1@saglik.gov.tr (C.Ü.)

**Keywords:** brain death, children, computed tomography, angiography

## Abstract

Background/Objectives: While ancillary tests for brain death diagnosis are not routinely recommended in guidelines, they may be necessary in specific clinical scenarios. Computed tomography angiography (CTA) is particularly advantageous in pediatric patients due to its noninvasive nature, accessibility, and rapid provision of anatomical information. This study aims to assess the diagnostic sensitivity of a revised venous system (ICV-SPV) utilizing a 4-point scoring system in children clinically diagnosed with brain death. Materials and Methods: A total of 43 pediatric patients clinically diagnosed with brain death who underwent CTA were retrospectively analyzed. Imaging was performed using a standardized brain death protocol. Three distinct 4-point scoring systems (A20-V60, A60-V60, ICV-SPV) were utilized to assess vessel opacification in different imaging phases. To evaluate age-dependent sensitivity, patients were categorized into three age groups: 26 days–1 year, 2–6 years, and 6–18 years. The sensitivity of each 4-point scoring system in diagnosing brain death was calculated for all age groups. Results: The revised venous scoring system (ICV-SPV) demonstrated the highest overall sensitivity in confirming brain death across all age groups, significantly outperforming the reference 4-point scoring systems. Furthermore, the ICV-SPV system exhibited the greatest sensitivity in patients with cranial defects. Conclusions: The revised 4-point venous CTA scoring system, which relies on the absence of ICV and SPV opacification, is a reliable tool for confirming cerebral circulatory arrest in pediatric patients with clinical brain death.

## 1. Introduction

Brain death, defined as the irreversible cessation of all brain functions, including the brainstem, is a well-established medical and legal diagnosis [1]. The clinical determination of brain death, first described in 1968 by a Harvard Medical School multidisciplinary committee and later codified in updated guidelines, remains the gold standard in both adult and pediatric populations. Clinically, brain death is diagnosed based on the presence of coma, brainstem areflexia, and apnea in response to an adequate stimulus [1,2,3].

Ancillary tests are recommended only in cases where clinical evaluation cannot be safely or completely performed, such as when apnea testing is contraindicated or incomplete [4,5]. Establishing clinical brain death criteria in pediatric patients is particularly challenging due to the immaturity of central venous systems, the incomplete development of brainstem reflexes in neonates younger than 37 weeks, hemodynamic instability, hypothermia, and difficulties in performing the apnea test. Consequently, there is a greater reliance on ancillary testing in pediatric populations [6].

Several ancillary tests are available, including transcranial Doppler ultrasound, electroencephalography (EEG), brain single-photon emission computed tomography (SPECT), magnetic resonance angiography (MRA), CTA, and conventional angiography. Among these modalities, CTA is particularly valuable in pediatric patients due to its noninvasive nature, widespread availability, and ability to rapidly provide anatomical information [7,8].

Despite its utility, there is no consensus on CTA evaluation protocols for brain death, and various studies have proposed different scoring systems [9]. To evaluate cortical and brainstem perfusion, the ideal CTA scoring system should assess blood flow in both the supratentorial and infratentorial regions [10]. One of the most widely used methods is the 4-point scoring system by Frampas et al. [11], which assesses contrast enhancement in the middle cerebral artery (MCA) M4 branches and internal cerebral veins (ICVs). However, this scoring system has limitations, particularly in patients with open fontanelles or craniectomies.

Recent studies have introduced a revised 4-point venous CTA scoring system, incorporating the superior petrosal vein (SPV) along with the ICV, to improve diagnostic accuracy [10,12]. This system has shown promising results in adult populations, particularly in patients with cranial defects. However, to date, no study has evaluated the diagnostic sensitivity of this revised venous system in pediatric brain death.

The present study aims to assess the diagnostic performance of the revised ICV-SPV scoring system in children with clinically confirmed brain death and to compare its sensitivity with previously established 4-point scoring systems.

## 2. Methods

### 2.1. Study Population

Children aged 3 months to 18 years who underwent computed tomography angiography (CTA) and were preliminarily diagnosed with brain death between October 2022 and May 2024 were retrospectively analyzed. This study included 43 patients under 18 years of age who were clinically diagnosed with brain death and underwent CTA as an ancillary test. Brain death was confirmed clinically in all patients before imaging. Patients were excluded if the venous phase could not be evaluated due to technical limitations or if contrast opacification was uninterpretable due to severe pseudosubarachnoid hemorrhage.

A total of 43 children (13 females, 30 males; age range: 3–216 months; median age: 84 months [IQR: 24–180]) met the inclusion criteria. All CTA examinations were technically sufficient for analysis.

The most common etiologies of brain death were traumatic brain injury and cardiac arrest. The demographic characteristics and causes of brain death are summarized in Table 1.

### 2.2. Preliminary Anatomical Study of Infratentorial Veins in Children

Marchand et al. conducted a preliminary anatomical study in adults to assess the infratentorial venous circulation and develop a revised 4-point venous CTA scoring system [10]. Similarly, we performed a preliminary study in pediatric patients to evaluate venous visibility using the same approach.

**Figure 1:**
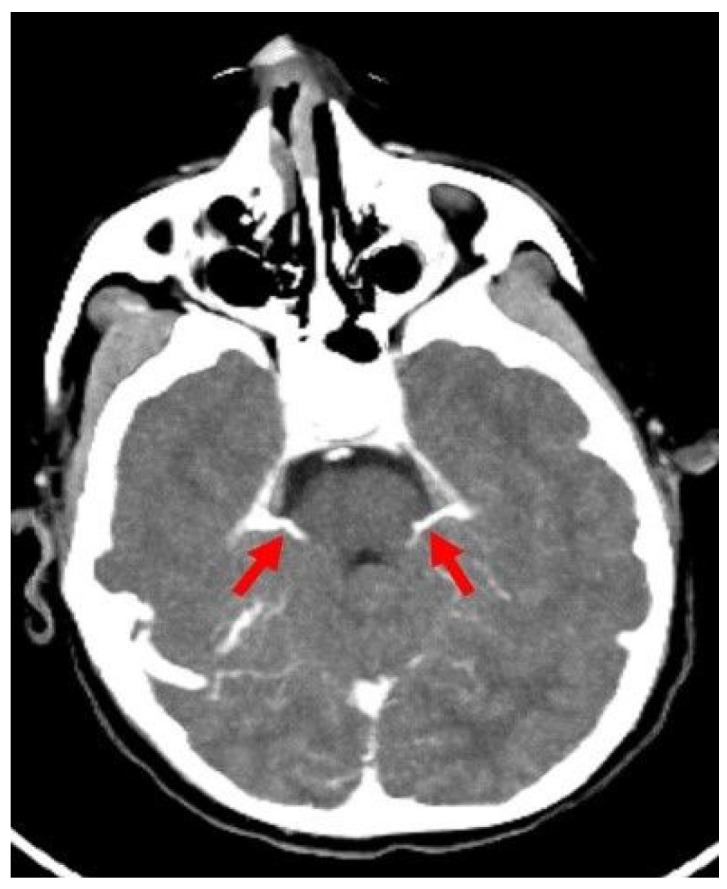
Axial CTA image of a control patient without brain death shows SPVs (red arrows) draining into the petrosal sinus at the level of the middle and superior cerebral peduncle of the mesencephalon. CTA: CT angiography, SPV: Superior petrosal vein.

For this study, 40 control patients (25 males, 15 females; mean age: 7.6 years) were classified based on the visibility of the internal cerebral vein (ICV) and superior petrosal vein (SPV). The veins were categorized as fully visible (normotrophic), hypotrophic but visible, or absent (Figure 1). The ICV was identifiable and normotrophic in all patients. The SPV was visible in all cases but was hypotrophic in 30% of subjects.

### 2.3. Imaging Acquisition

All examinations were performed using a 128-slice CT scanner (GE Revolution EVO, Chicago, IL, USA). The scanning parameters were 100 kVp, 200 mAs, 0.6 mm slice thickness, 0.55 mm interval, 220 mm field of view, and a 512 × 512 m^2^ matrix. All patients were hemodynamically stable.

CTA was performed 24 h a day for babies between 2 months and 1 year old and 12 h a day for children over 1 year old after cardiocirculatory arrest.

**Figure 2:**
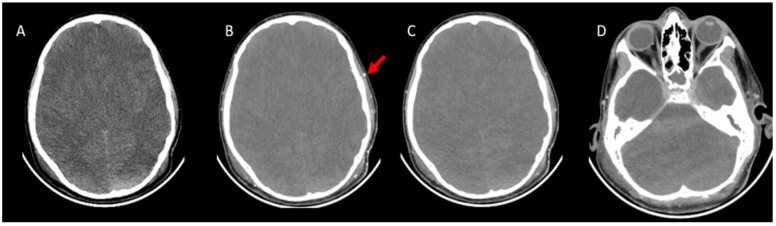
CTA images were taken with the brain death protocol of a 13-year-old boy who was brain dead. (**A**), Axial pre-contrast CT image shows cerebral edema with the disappearance of the ventricles and flattening of the subarachnoid sulci. (**B**), Axial post-contrast arterial-phase (20th second) image shows opacification of superficial temporal artery (red arrow), which indicates adequate technique. (**C**), Axial post-contrast venous-phase image (60th second). The arterial phase and venous phase show an absence of opacification of the bilateral MCA and branches. (**D**), The venous phase shows the absence of opacification of the ICV and SPV. CTA: CT angiography, MCA: Middle cerebral artery, ICV: Internal cerebral vein, SPV: Superior petrosal vein.

The brain parenchyma was scanned from the convexity level to the C1-C2 level with a slice thickness of 1 mm in three phases (Figure 2). CTA scans were acquired with the brain death protocol consisting of an unenhanced scan, arterial phase scan at the 20th second, and venous phase scan at the 60th second. After the unenhanced images were acquired, 2 mg/kg of intravenous (IV) nonionic iodinated contrast medium (Iohexol; Opaxol, 300 mg iodine/mL, Opakim) was administered by injecting at a rate of 3 mL/s.

CTA evaluations were performed using axial thin-section images, Multiplanar Reconstruction (MPR), and Maximum Intensity Projection (MIP) images.

### 2.4. Image Interpretation

Non-contrast images were evaluated for diffuse brain edema, sulcal and tonsillar herniation, subarachnoid and intraventricular hemorrhage, cranial bone integrity, anterior fontanelle patency, and the presence of shunts. Cranial bone integrity was classified as compromised in cases of open fontanelles, decompressive craniectomy, shunt defects, or calvarial fractures.

The accuracy of the imaging technique was confirmed by assessing the normal opacification of the superficial temporal arteries during the arterial phase. All CTA scans were independently reviewed by two radiologists (H.G.Ç. and Ş.Y.), each with at least 10 years of experience in CTA interpretation, who were blinded to each other’s findings.

The CT angiograms were subsequently re-evaluated using three different 4-point scoring systems to assess the opacification of four vessels across different imaging phases. The 4-point scoring systems analyzed were the following:

1. A60-V60: Absence of opacification in M4 branches of the middle cerebral arteries (MCAs) and ICVs in the venous phase, based on the reference 4-point system by Frampas et al. [11].
2. A20-V60: Absence of opacification in M4 branches of the MCAs in the arterial phase and ICVs in the venous phase, based on the revised arteriovenous scoring system by Nunes et al. [12].
3. ICV-SPV: Absence of opacification in the ICVs and SPVs in the venous phase, based on the revised venous scoring system by Marchand et al. [10].

Each non-opacified vessel segment was assigned 1 point. The findings on CTA were interpreted as indicative of brain death only if a score of 4 was achieved in any of the scoring systems, indicating the absence of opacification in all evaluated vessels.

To determine the age-dependent sensitivity of CTA, children were divided into three groups: 26 days–1 year, 1–6 years, 6–18 years. The sensitivities of the 4-point scoring systems for diagnosing brain death were calculated for all age groups.

Brain death was confirmed with scintigraphy in 5 patients.

### 2.5. Statistical Analysis

Statistical analysis was performed using SPSS version 26.0 (Armonk, NY, USA: IBM Corp). The Kolmogorov–Smirnov test was used to assess the normality of data distribution. For qualitative data, numbers and percentages are presented, and for quantitative data, the mean and standard deviation are used if normally distributed; otherwise, the median and interquartile ranges are reported.

The sensitivity of the 4-point scoring systems and the revised venous system in patients clinically diagnosed with brain death was calculated. Sensitivity was defined as the percentage of brain death cases accurately confirmed by the imaging method and criteria. McNemar’s test was used to compare the sensitivities of the A20-V60 and A60-V60 4-point scoring systems. The evaluation of opacification in craniectomized versus non-craniectomized patients was assessed using the Chi-square test. The inter-observer correlation of sensitivities was analyzed using kappa statistics. Kappa values, ranging from 0 to 1, were classified as follows: <0.20, poor agreement; 0.21–0.40, fair agreement; 0.41–0.60, moderate agreement; 0.61–0.80, good agreement; and 0.81–1.00, excellent agreement. A *p*-value < 0.05 was considered statistically significant.

## 3. Results

**Figure 3:**
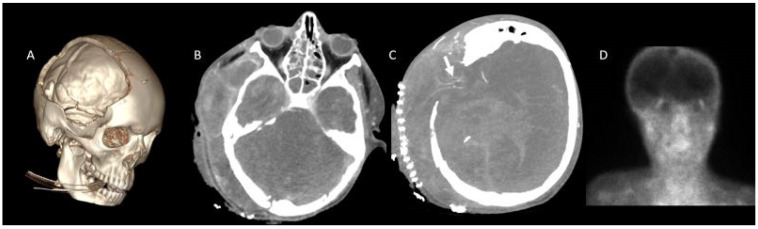
CTA in a 13-year-old boy with clinically confirmed brain death. (**A**), Craniectomy defect is visualized in VR images. (**B**), A lack of opacification is observed in the SPV and ICV during the venous phase. (**C**), However, persistent opacification of the right MCA-M4 segment is noted (white arrow). (**D**), Brain perfusion was absent on scintigraphy, further supporting the diagnosis of brain death. CTA: CT angiography, MCA: Middle cerebral artery, ICV: Internal cerebral vein, SPV: Superior petrosal vein, VR: 3D volume-rendered.

**Figure 4:**
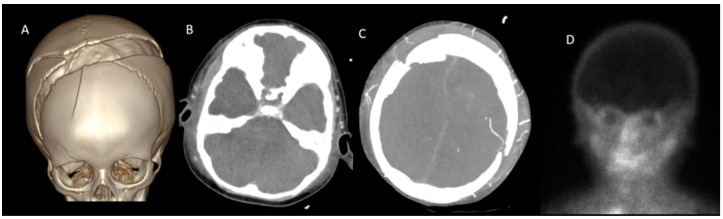
CTA in a 2-year-old boy with clinically confirmed brain death. (**A**), A cranial fracture is visualized in VR images. (**B**), A lack of opacification is observed in the SPV and ICV during the venous phase. (**C**), However, persistent opacification of the right MCA-M4 segment is noted (white arrow). (**D**), Brain perfusion was absent on scintigraphy, further supporting the diagnosis of brain death. CTA: CT angiography, MCA: Middle cerebral artery, ICV: Internal cerebral vein, SPV: Superior petrosal vein, VR: 3D volume-rendered.

Patients were classified based on the presence or absence of cranial bone defects (Table 2). Among the 43 children, 24 had intact cranial bone structures, while 19 exhibited cranial bone defects (Figure 3 and Figure 4).

On non-contrast CT, diffuse brain edema with accompanying tonsillar herniation was observed in 40 patients, while 3 patients did not exhibit diffuse brain edema. Additionally, diffuse brain edema was associated with subarachnoid hemorrhage in 6 patients and with intraventricular hemorrhage in 10 patients (Table 3).

Interobserver reliability was excellent for the A20-V60 scoring system (97.6%, κ = 0.909). It was moderate for the A60-V60 (95.3%, κ = 0.927) and ICV-SPV (93%, κ = 0.876) scoring systems. The interobserver agreement levels for the scoring systems are listed in Table 4.

Stasis filling was detected in 15 patients (34%), of whom 6 presented with cranial defects (e.g., open fontanelles, craniectomy, shunt, or fracture). However, the association between cranial defects and stasis filling was not statistically significant (*p* = 0.685).

**Figure 5:**
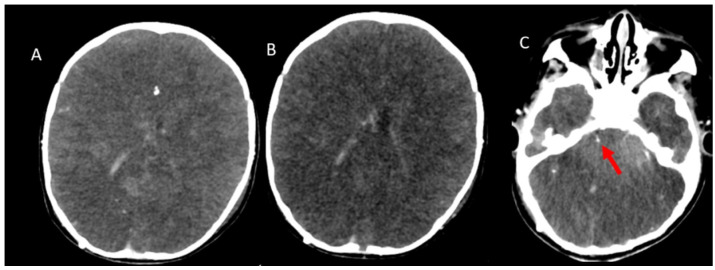
CTA was performed in a 1-year-old boy with clinically confirmed brain death who did not meet the criteria for brain death. (**A**), Arterial-phase image and (**B**,**C**), Venous-phase images reveal an absence of opacification in the branches of the MCAs. Conversely, opacification of the SPV is seen (red arrow). CTA: CT angiography, MCA: Middle cerebral artery, ICV: Internal cerebral vein, SPV: Superior petrosal vein.

In our study, while evaluating the sensitivities of the A20-60 and A60-60 systems and the revised venous system (ICV-SPV), children were divided into three groups: 29 days–1 year, 1–6 years, and 6–9 years. The ICV-SPV system exhibited the highest overall sensitivity (88.4%) in confirming brain death across all age groups (Table 5, Figure 5). In children aged 6–18 years, the ICV-SPV system demonstrated a sensitivity of 86.3%, surpassing that of the other systems. The A20-V60 system exhibited the highest sensitivity (95.5%) in infants aged 29 days to 1 year, whereas the A60-V60 system showed the greatest sensitivity (92.3%) in children aged 1–6 years. Furthermore, the revised 4-point venous scoring system demonstrated significantly higher overall sensitivity, as well as greater sensitivity in the 6–18-year age group, compared to the reference 4-point scoring system (A60-V60) (*p* = 0.001).

In patients with cranial defects, the sensitivity of all scoring systems was lower compared to those with intact cranial structures. In patients with cranial defects, the diagnostic sensitivity of all evaluated scoring systems was diminished relative to that observed in patients with intact cranial structures. Notably, the ICV-SPV scoring system demonstrated the highest sensitivity at 89.5%; however, this difference did not reach statistical significance (Table 6, Figure 3 and Figure 4).

Brain death was confirmed by scintigraphy in four of the five patients in whom this ancillary test was performed.

## 4. Discussion

CT angiography (CTA) has been extensively studied as an ancillary method for confirming brain death, primarily in adult populations [13,14,15]. Previous research has investigated the advantages of CTA and compared the sensitivities of various scoring systems [16,17,18]. However, most of the literature has focused on adult patients. Eda et al. evaluated the sensitivities of 10-point, 7-point, and 4-point scoring systems for diagnosing brain death in pediatric patients [19]. To our knowledge, the present study represents the second investigation into the use of CTA for pediatric brain death diagnosis.

Prior studies have demonstrated that 4-point scoring systems exhibit superior sensitivity compared to those incorporating a greater number of vascular structures [20,21,22]. Furthermore, Marchand et al. introduced the revised 4-point venous scoring system (ICV-SPV) in adults, which demonstrated high sensitivity for confirming brain death [10]. The present study aimed to assess the sensitivity of this revised system in pediatric patients in comparison with the 4-point systems previously described by Frampas [11] and Nunes [12]. To our knowledge, this is the first study to evaluate the revised system specifically in children.

Frampas et al. emphasized the importance of venous circulation in brain death diagnosis, demonstrating that the absence of internal cerebral vein (ICV) opacification was more sensitive than the absence of cortical middle cerebral artery (MCA) opacification [11]. Based on the premise that the infratentorial venous system plays a critical role in evaluating brainstem viability in brain death, the revised venous system was designed to assess supratentorial circulation via the ICV and infratentorial circulation via the superior petrosal vein (SPV).

In the present study, the revised venous system exhibited a sensitivity of 88.4%. Although this sensitivity was lower than that reported in adults (95.4%), it remained higher than that of other scoring systems (A20-V60, A60-V60) [10,23]. These findings indicate that the revised venous system is a sensitive method for diagnosing brain death in pediatric patients.

Notably, the sensitivities of all evaluated scoring systems were lower in pediatric patients compared to adults. This discrepancy may be attributed to anatomical and physiological differences in vascular structures between children and adults. Studies have consistently shown that the absence of ICV opacification is the most sensitive criterion for brain death diagnosis. This observation aligns with Asgeirsson et al.’s hypothesis that “in an organ surrounded by a rigid partition, an increase in tissue pressure does not affect arterial resistance but instead leads to increased venous resistance [10,23].

As presented in Table 5, the sensitivity rates varied across the evaluated systems depending on age. In children aged 6–18 years, the ICV-SPV system demonstrated a high sensitivity of 86.3%, outperforming the other systems within this age group. In contrast, the A20-V60 system exhibited the highest sensitivity (95.5%) in infants aged 29 days to 1 year, while the A60-V60 system achieved a sensitivity of 92.3% in children aged 1–6 years. Moreover, the 6–18-year age group comprised the largest sample size (*n* = 22), which is more than twice the number of subjects in the other groups. This discrepancy suggests that the differences in sample sizes across age groups may have contributed to inhomogeneity in the sensitivity assessments.

In patients with cranial defects, the sensitivities of all scoring systems were lower compared to those with intact cranial structures, although this difference was not statistically significant. Similar to findings in adult studies, the ICV-SPV system demonstrated high sensitivity for diagnosing brain death in craniectomized pediatric patients [10,23]. These findings underscore the importance of utilizing the ICV-SPV scoring system in such cases.

Although the sensitivity was reduced in patients with cranial defects, as observed in previous studies [19,20], the A20-V60 method exhibited a significantly higher sensitivity than the A60-V60 method in this subgroup (100% vs. 83.3%, respectively).

The interobserver agreement was excellent for the A20-V60 method but moderate for the A60-V60 and ICV-SPV methods. The relatively lower agreement in venous phase scoring may be attributed to the presence of pseudosubarachnoid hemorrhage, which complicates the assessment of vascular opacification. Some studies have proposed the use of subtraction techniques to mitigate this challenge [13].

In this preliminary study, ICV assessment was found to be readily feasible in CTA examinations performed for various indications. While some studies have excluded SPV assessment due to its perceived difficulty, our findings highlight the importance of evaluating the SPV in brain death diagnosis. A thorough understanding of its anatomy and imaging characteristics can facilitate its accurate assessment.

The primary limitation of this study was the absence of a control group, which precluded an evaluation of the specificity of CTA for brain death diagnosis. Additionally, brain death was confirmed using scintigraphy in only five patients, limiting direct comparisons with other confirmatory tests. Furthermore, some patients exhibited pseudosubarachnoid hemorrhage findings. The assessment of vascular opacification in these cases has been challenging due to the unavailability of subtraction imaging at our institution. To address this limitation, an alternative approach was employed by simultaneously evaluating precontrast and postcontrast images on the same screen. Finally, we classified the study participants into three age-based groups. However, the inhomogeneity observed among these groups constitutes another limitation of our study.

In conclusion, the revised 4-point venous CTA scoring system, based on the absence of ICV and SPV opacification, is a reliable method for confirming cerebral circulatory arrest in pediatric brain death. It demonstrates superior diagnostic performance compared to previously established 4-point CTA scoring systems.

## 5. Conclusions

Assessing SPV opacification may initially appear challenging, but we underline its importance in diagnosis and encourage its inclusion in evaluations. This study is, to our knowledge, only the second to assess CTA in pediatric brain death diagnosis and highlights the need for further research involving larger control groups and comparative analyses of different scoring systems.

## Figures and Tables

**Table 1:** Causes of brain death and demographic characteristics of patients (*n* = 43).

Variable	Number (%)
Sex	
Male	30 (69.8)
Female	13 (30.2)
Etiology of brain death	
Trauma	12 (27.9)
Cardiac arrest	12 (27.9)
Asphyxia	9 (20.9)
İnfection	5 (11.6)
İntoxication	2 (4.7)
Metabolic disease	2 (4.7)
Terminal stage tumor	1 (2.3)

**Table 2:** Calvarial bone defects of patients.

Variable	*n*/%
Open Fontanel	12 (27.9)
Craniectomy	2 (4.7)
Fracture	5 (11.6)
Shunt	5 (11.6)

**Table 3:** Non-contrast CT findings in brain death.

Variable	*n*/%
DCE + TH	40 (93)
SAH	6 (13)
IVH	10 (23.3)

DCE + TH: Diffuse cerebral edema with tonsiller herniation, SAK: Subarachnoid hemorrhage, IVH: Intraventricular hemorrhage.

**Table 4:** Interobserver agreement and kappa values for scoring systems of CT angiography.

Scoring System	Agreement for Diagnosis of Brain Death	
	κ	%
A20-V60	0.909	97.6
A60-V60	0.927	95.3
ICV-SPV	0.876	93.0

**Table 5:** Sensitivities of the 4-point scoring systems (A20-V60, A60-V60, SPV-ICV) in children.

	OS	29 d < 1 y (*n*:8)	1–6 y (*n*:6)	>6 y (*n*:22)
CTA (%)				
A20-V60	86.0	95.5	84.6	81.8
A60-V60	79.1	87.5	92.3	81.8
ICV-SPV	88.4	87.5	84.6	86.3

OS: Overall sensitivity, ICV: Internal cerebral vein, SPV: Superior petrosal vein.

**Table 6:** Sensitivities of the 4-point scoring systems (A20-V60, A60-V60, SPV-ICV) in children with or without open skull defects.

	OSD+	OSD−	*p*
CTA (%)			
A20-V60	84.2	87.1	1.0
A60-V60	68.4	86.2	0.477
ICV-SPV	89.5	87.8	1.0

OSD: open skull defect, ICV: Internal cerebral vein, SPV: Superior petrosal vein, *p* < 0.05 was considered statistically significant.

## Data Availability

The data presented in this study are available upon request from the corresponding authors. The data are not publicly available due to privacy.
